# CyberKnife Stereotactic Radiosurgery for Primary and Metastatic Cancer in the Cervix

**DOI:** 10.7759/cureus.2002

**Published:** 2017-12-29

**Authors:** Yuko Harada, Shinichiro Miyazaki

**Affiliations:** 1 Internal Medicine, Shin-yurigaoka General Hospital; 2 Radiation Oncology, Shin-yurigaoka General Hospital

**Keywords:** cervical cancer, cyberknife, sbrt

## Abstract

Standard radiation therapy for cervical cancer consists of external beam radiation therapy followed by an intracavitary brachytherapy boost. When brachytherapy cannot be performed due to unfavorable anatomy or coexisting medical conditions, stereotactic body radiotherapy with the CyberKnife is another treatment option which is less invasive and can be performed in a shorter treatment time. We performed non-invasive therapy using the CyberKnife for five cases of urogenital cancer located in the cervix. The local tumor control was excellent with minimal toxicity. Non-invasive treatment was demonstrated as feasible with the CyberKnife for contraindication of surgery or brachytherapy.

## Introduction

Brachytherapy (BT) boost supplementing concurrent chemotherapy and external beam radiation therapy (EBRT) have been the standard therapy for locally advanced cervical cancer [1]. However, complications of BT arise from inappropriate placement in a technique that is sensitive to physician skills. Numerous patients are excluded from BT due to physical conditions which prevent applicator placement, such as decreased vaginal accommodation with age, uterine malformations, or excessive tumor volume [2].

The other urogenital cancers, such as endometrial cancer and bladder carcinoma, can metastasize in the cervix, which is located in the center of pelvis. Standard radiation therapy is difficult due to the depth and the surrounding organs. Stereotactic body radiotherapy (SBRT) or BT are the options of treatment for inoperable cancer.

The CyberKnife system (Accuray Incorporated, Sunnyvale, California) is a robotic radiosurgery system that offers highly precise SBRT. Its robotic precision and accuracy provide much shorter treatment times and superior performance. We performed SBRT on five patients with primary or metastatic cancer in the cervix utilizing the CyberKnife.

## Materials and methods

Five patients were treated with the CyberKnife for cervical lesions since April 2015 as shown in Table 1. The primary diseases consisted of three cases of cervical cancer (one untreated, one relapse after surgery, and one relapse after surgery and radiation therapy), one case of endometrial cancer, and one case of bladder cancer. The primary treatments were surgery, surgery followed by radiation therapy, and chemotherapy followed by surgery. The median age was 67 years old (64 to 84) and median time to recurrence from initial diagnosis was 70.5 months (7 to 228 months). The median follow-up duration was 12 months (four to 24 months).

**Table 1:** Characteristics, treatment plans, and outcome of the five cases. GTV: gross tumor volume. PTV: planning target volume. Scc: squamous cell carcinoma. RALS: remote after loading system. PDT: photodynamic therapy. CR: complete remission. PR: partial remittion. PD: progressive disease.

CASE	1	2	3	4	5
AGE	78	65	84	67	64
PRIMARY CANCER	Cervical Cancer	Cervical Cancer	Cervical Cancer	Endometrial Cancer	Bladder Cancer
HISTOLOGY	Scc	Scc	Scc	endometrioid adenocarcinoma	Urothelial Carcinoma
STAGE	ⅡA2	ⅡA1	ⅡB	ⅠB	Ⅲ
SURGERY	none	Total Hysterectomy	Total Hysterectomy	Total Hysterectomy	Radical Cystectomy
CHEMOTHERAPY	none	none	none	none	Gemcitabine+ Cisplatin
OTHER TREATMENT PRIOR TO SBRT	N/A	N/A	RALS+PDT	N/A	N/A
TIME TO RECURRENCE FROM DIAGNOSIS (months)	N/A	26	228	115	7
FRACTIONS	10	8	10	7	10
PRESCRIPTION DOSE FOR CERVICAL TUMOR (cGy)	4500	4400	2500	4200	3538
GTV (cm^3^)	120.9	2.5	71	32	87.5
PTV (cm^3^)	205.6	9.8	75.7	62.7	157.4
D95 ISODOSE (%)	63	77	60	60	55
OUTCOME	12 months CR	6 months CR	14 months PD	4 months PR	6 months CR
SECOND SBRT(GTV, PRESCRIPTION DOSE)	N/A	N/A	14.7cm^3^, 3500cGy	N/A	N/A

All the patients had positron emisson tomography and comupted tomography (PET/CT) scans to evaluate the entire body prior to SBRT. Vac-Lok^TM^ Cushon (CIVCO, Coralville, Iowa) was prepared for individual positioning. Tumors were tracked with X-site spine tracking algorithm. The gross tumor volume (GTV) was defined as visible tumor on enhanced CT and PET/CT with images merged for target definition. GTV was considered the same as clinical target volume (CTV). The planning target volume (PTV) included the CTV and a margin of 2 mm. Vac-Lok^TM^ Cushon was used for positioning during SBRT.

The GTV ranged from 2.5 cm^3^ to 120.9 cm^3^ (mean 62.8 cm^3^, median 72.0 cm^3^). The prescription dose was 25 to 45 Gy (median 42 Gy). The fraction ranged from 7 to 10 (mean 9). D 95 isodose ranged from 55 to 77% (mean 63%).

All the patients were monitored for one, two, three, four, and six months after SBRT with physical examination and blood tests. They had follow-up CT scans in two months, follow-up PET/CT scan in four or six months, and follow-up CT or PET/CT scan every six months thereafter to evaluate the result of SBRT. The median follow-up was six months (four to 12 months). Local recurrence was defined as a lesion that developed within PTV. A tumor that appears outside the radiation target is defined as distant failure.

**Figure 1:**
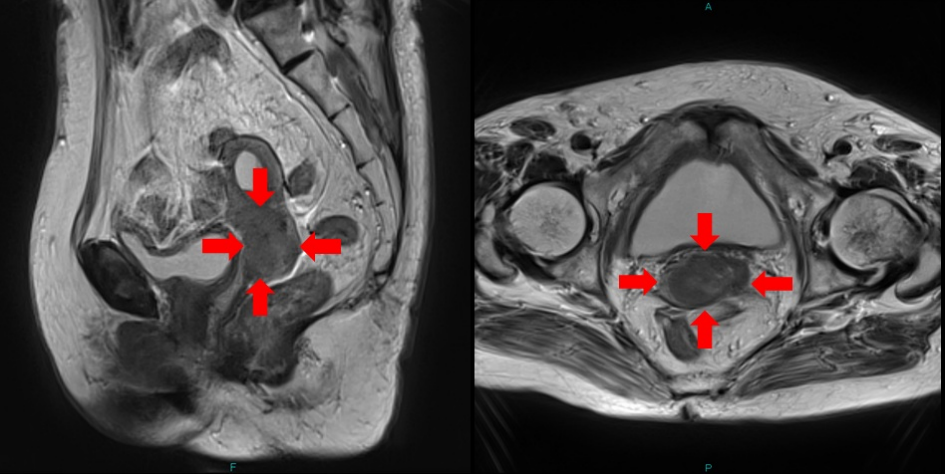
MRI of Case 1. T2WI (T2 weighted image) of Case 1 before SBRT. The tumor is shown by the red arrows. (MRI: magnetic reasonance imaging)

**Figure 2:**
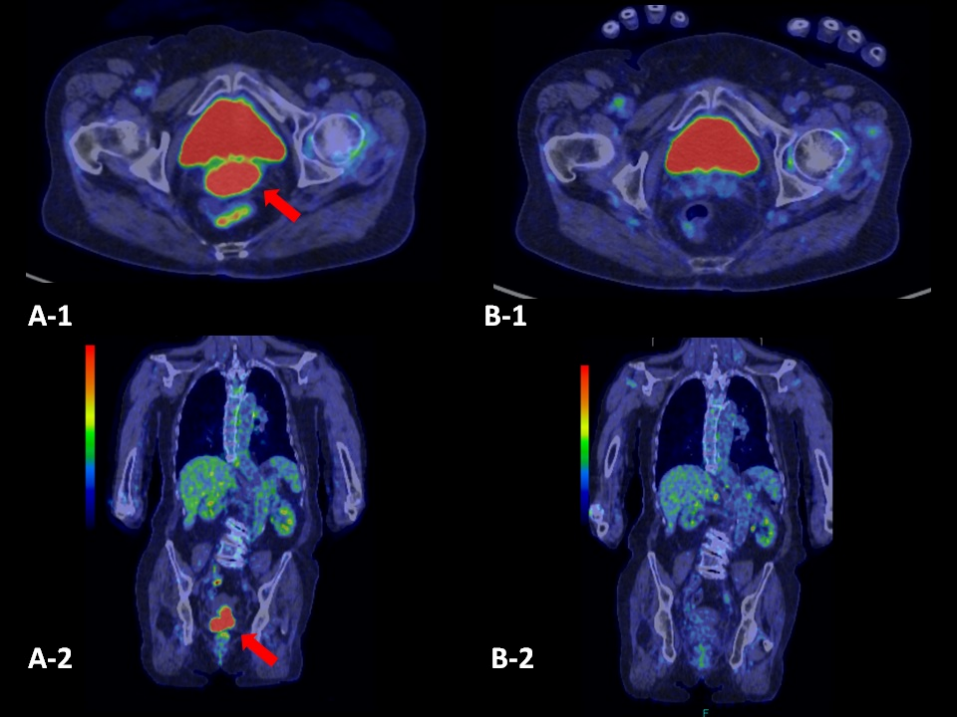
FDG (fluorodeoxyglucose)-PET of Case 1. A-1, 2: before SBRT. The FDG-uptake is shown in the cervix (the red arrows). B-1,2: 12 months after SBRT. The tumor has disappeared. (PET: positron emission tomography)

**Figure 3:**
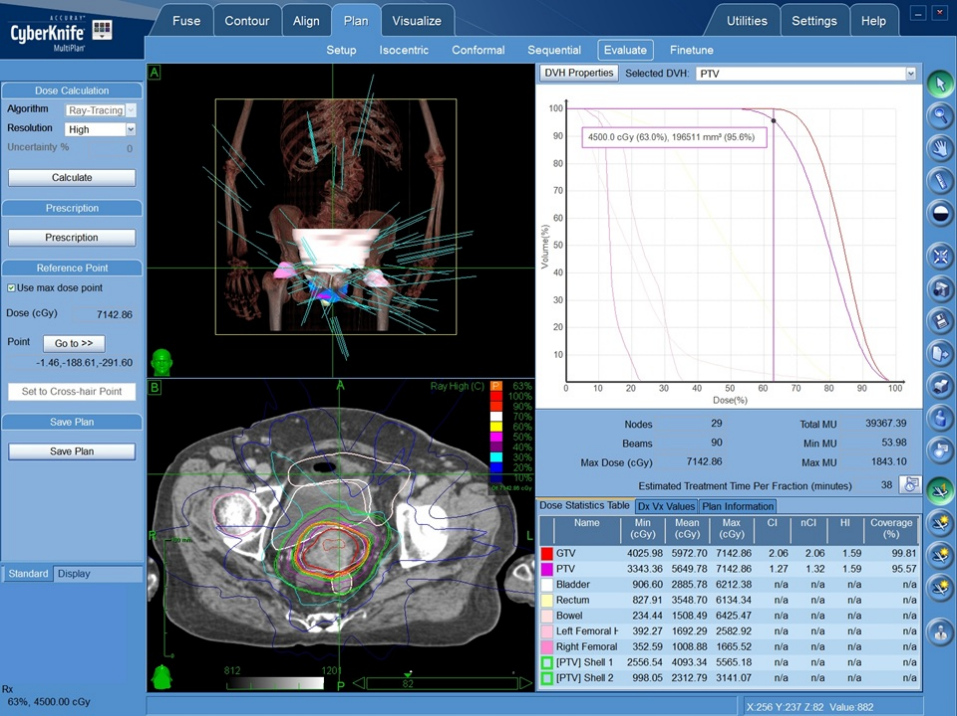
CyberKnife treatment plan of Case 1.

**Figure 4:**
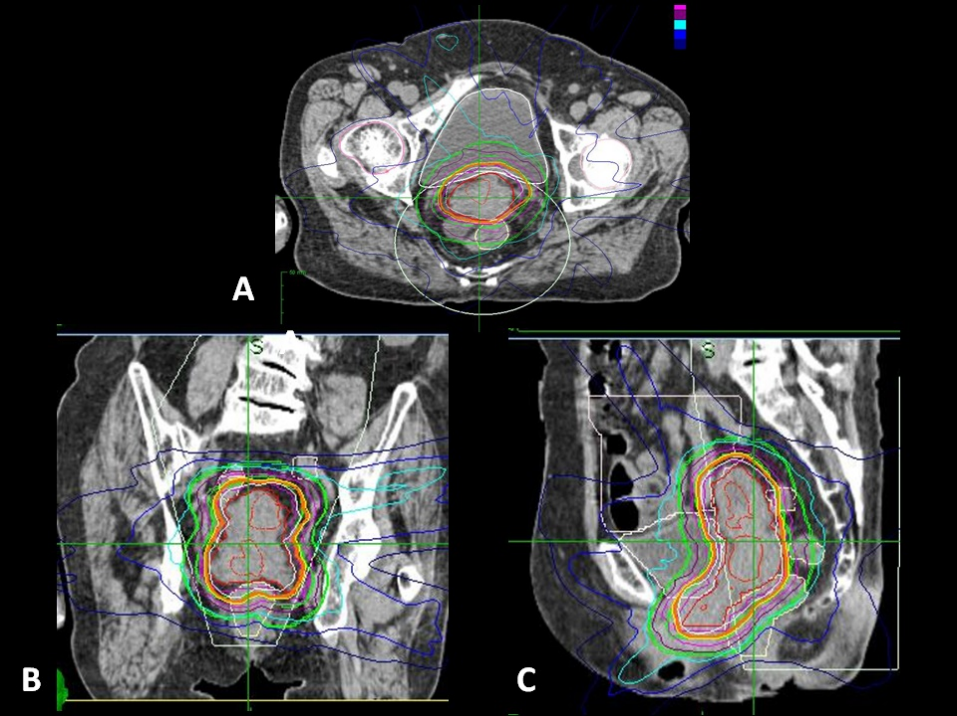
CyberKnife planning process of Case 1. Axial (A), coronal (B), and sagital (C) images of the planning CT. The tumor is contoured, and the volume and radiation doses are calculated accordingly. (CT: computed tomography)

Taking Case 1 for example, the magnetic reasonance imaging (MRI) image of the tumor is shown in Figure 1, and the PET/CT before and after SBRT is shown in Figure 2. CyberKnife treatment plans are shown in Figure 3. CyberKnife planning process is shown in Figure 4.

## Results

Three patients achieved complete remission (CR), and two patients achieved partial remission (PR) as shown in Table 1. Only one case (Case 3) showed progressive disease (PD) and underwent a second SBRT.

PET/CT scan at 12 months indicated that Case 3 in fact achieved PR. The follow-up CT two months later, however, identified a new and separate tumor in the cervix which is defined as distant failure. The patient underwent a second SBRT with 35 Gy in five fractions for the new and separate tumor with GTV of 14.7 cm^3^. Seven months later she developed rectal-vaginal fistula, which is Grade 4 in Common Terminology Criteria for Adverse Events.

The other four cases did not have any radiation toxicity. No local recurrence was observed during the follow-up period.

## Discussion

SBRT with the CyberKnife was very successful in four cases of primary and metastatic cancer in the cervix. Only Case 3 had local recurrence due to intentional small prescription dose considering her history of previous radiation therapy and laser treatment. After the second SRBT, she developed rectal-vaginal fistula. Since she was an elderly patient with a lengthy disease history of 18 years plus with multiple radiation and laser treatments, such complication or relapse was inevitable.

The prescription dose and treatment plan for CyberKnife treatment were determined considering multiple factors, such as tumor volume, the number of fraction, collimator size, D95 isodose, previous radiation therapies, and critical consideration of adjacent organs. The radiation doses were calculated as 2-Gy equivalent doses using the linear quadratic model with α/βratio of 3 Gy for bladder and rectum and α/βratio of 10 Gy for the tumor. Using α/βratio of 10 Gy, the prescription doses of Case 1 to 5 were roughly 54 Gy, 56 Gy, 26 Gy, 50 Gy, and 40 Gy, respectively. Thus, we used small dose for the first SBRT of Case 3, but the dose for the second SBRT was much larger (calculated to 50 Gy using α/βratio of 10Gy), which led to Grade 4 radiation toxicity. Even with the CyberKnife, it was still difficult to treat the cases with previous and multiple radiation therapies.

Pelvic cancer, irrelevant of primary, recurrent or metastasis, is difficult to treat. Gill, et al. reported that after controlling for significant factors from survival analyses, IMRT or SBRT boost resulted in inferior overall survival (hazard ratio, 1.86; 95% confidence interval, 1.35-2.55; P<.01) as compared with brachytherapy [3]. However, several studies have reported good results with SBRT [4-8]. Guckenberger, et al. reported 19 cases of recurrent cervical cancer or endometrial cancer had 81% local control in three years and 59% Median survival in two years with SBRT [7]. Deodato, et al. reported SBRT in recurrent gynecological cancer reached CR in 66 % of the cases and overall survival in two years was 63.6% [8]. Dewas, et al. reported that SBRT with the CyberKnife provided a short and well-tolerated treatment for lateral pelvic recurrences in previously irradiated areas irrelevant of the history of the primary cancer [9]. These studies showed that SBRT is in fact a favorable alternative treatment for pelvic cancer.

Hasan, et al. compared CyberKnife treatment for recurrent gynecological malignancies in the central pelvis, pelvic sidewall, and para-aortic lymph node lesions, and reported that local control and prognosis were poor in the central pelvis [10]. Although this was a small study with 30 patients, it demonstrated that tumors in the central pelvis (such as cervical cancer) are difficult to treat even with the CyberKnife.

In the literature there are two ways to use the CyberKnife to treat cancer in the cervix: to utilize the CyberKnife in place of BT as a boost after EBRT [11-13], or to use the CyberKnife alone [10]. However, it is standard practice to implant three or four gold fiducial markers for tracking the tumor, which is invasive and oftentimes intolerable for the patients. Muacevic, et al. reported that fiducial-free spinal tracking of the lower lumbar vertebrae is a feasible, accurate, and reliable tool for radiosurgery of sacral and pelvic tumors [4]. We followed Muacevic’s procedure to treat the cancer in the cervix, and the results were satisfactory during the follow-up period. It is thus demonstrated far more sensible to use the CyberKnife without invasive procedures such as with BT or fiducial implantation, as the advantage of the CyberKnife is plain and simply its non-invasiveness.

In our study, the CyberKnife was used for SBRT to treat tumor in the cervix without EBRT and without implanting any fiducial marker, and obtained favorable results with less toxicity. A far simpler, far shorter, and fully non-invasive treatment was demonstrated to be feasible with the CyberKnife. The efficacy and toxicity need to be evaluated over the long term, but the CyberKnife is demonstrated a favorable treatment option for patients who cannot tolerate standard invasive therapy.

## Conclusions

CyberKnife stereotactic radiosurgery was shown successful in primary and metastatic cancer in the cervix. It thus provided non-invasive treatment with excellent local control and minimal toxicity.

## References

[1] Viswanathan AN, Thomadsen B, American Brachytherapy Society Cervical Cancer Recommendations Committee (2012). American Brachytherapy Society consensus guidelines for locally advanced carcinoma of the cervix. Part 1: general principles. Brachytherapy.

[2] Marnitz S, Kohler C, Budach V (2013). Brachytherapy-emulating robotic radiosurgery in patients with cervical carcinoma. Radiat Oncol.

[3] Gill BS, Lin JF, Krivak TC (2014). National Cancer Data Base analysis of radiation therapy consolidation modality for cervical cancer: the impact of new technological advancements. Int J Radiat Oncol Biol Phys.

[4] Muacevic A, Drexler C, Kufeld M, Romanelli P, Duerr HJ, Wowra B (2009). Fiducial-free real-time image-guided robotic radiosurgery for tumors of the sacrum/pelvis. Radiother Oncol.

[5] Wulf J, Hadinger U, Oppitz U, Thiele W, Flentje M (2004). Stereotactic boost irradiation for targets in the abdomen and pelvis. Radiother Oncol.

[6] Timmerman RD, Kavanagh BD, Cho LC, Papiez L, Xing L (2007). Stereotactic body radiation therapy in multiple organ sites. J Clin Oncol.

[7] Guckenberger M, Backmann J, Wulf J (2010). Stereotactic body radiotherapy for local boost irradiation in unfavourable locally recurrent gynaecological cancer. Radiother Oncol.

[8] Deodato F, Macchia G, Grimaldi L (2009). Stereotactic radiotherapy in recurrent gynecological cancer: a case series. Oncology Reports.

[9] Dewas S, Bibault JE, Mirabel X (2011). Robotic image-guided reirradiation of lateral pelvic recurrences: preliminary results. Radiat Oncol.

[10] Hasan S, Ricco A, Jenkins K (2016). Survival and control prognosticators of recurrent gynecological malignancies of the pelvis and para-aortic region treated with stereotactic body radiation therapy. Front Oncol.

[11] Haas JA, Witten MR, Clancey O, Episcopia K, Accordino D, Chalas E (2012). CyberKnife boost for patients with cervical cancer unable to undergo brachytherapy. Front Oncol.

[12] Podder T, Fried D, Holland B, Rosenman J, Biswas T (2012). SU-E-T-412: Can Cyberknife SBRT be an alternative to brachytherapy for cervical cancer treatment ?. Med Phys.

[13] Marnitz S, Kohler C, Budach V (2013). Brachytherapy emulatingrobotic radiosurgery in patients with cervical carcinoma. Radiat Oncol.

